# The role of prefrontal cortex and temporoparietal junction in interpersonal comfort and emotional approach

**DOI:** 10.1038/s41598-023-48099-0

**Published:** 2023-12-07

**Authors:** Vahid Nejati, Aylin Mardanpour, Abbas Zabihzaheh, Reza Estaji, Zahra S. Vaziri, Shahriar Shahidi

**Affiliations:** Department of Psychology, Shahid Beheshti University, Tehran, Iran

**Keywords:** Neuroscience, Psychology

## Abstract

Our perception of physical distance to individuals and stimuli is influenced by our mental distance and relatedness. The present study aimed to investigate the role of the dorsolateral prefrontal cortex (dlPFC), ventromedial prefrontal cortex (vmPFC), and right temporoparietal junction (rTPJ) in interpersonal comfortable distance and approach behaviors towards emotional stimuli. Twenty healthy volunteers received brain stimulation in four separate sessions with a one-week interval, including anodal left dlPFC, anodal right vmPFC, anodal rTPJ, and sham condition, with an extracranial return electrode. Our results revealed an increase in interpersonal distance during anodal rTPJ stimulation and a decrease in distance to positive pictures during anodal vmPFC stimulation. These findings suggest that the rTPJ plays a role in the perceptual component of self-other distancing, while the vmPFC is involved in approaching positive emotions.

## Introduction

Social cognition is the ability to understand and navigate social interactions based on representations of the self and others^1^. These two components, self and other, are essential for social cognition^2^. Most of our social processing involves these components and their interaction, including self-referential processing, theory of mind, in-group/out-group categorization, interpersonal relationships, and empathy. Although the interaction and interdependence of self and other are crucial for normal social processing, it is equally important to distinguish between these two components as an integral part of this interaction. Self-other discrimination (SOD) refers to the ability to determine the boundary of self against other at both physical and conceptual/mental levels. This distinction has been described as a prerequisite of higher social cognitive functions such as empathy^3,4^ and theory of mind^5^.

The SOD concept pertains to the demarcation and differentiation of self and others. Studies have shown that romantic relationships often involve blurred self and other boundaries and decreased interpersonal distance^6,7^, described in the metaphorical expression of “we are one”. Beyond this extreme integration, interpersonal distance serves as a social signal for approach/avoidance in our social interaction^8^. Different comfortable interpersonal distance has been described as a hallmark of different personalities^9,10^, attachment styles^11,12^, and psychopathologies^13–15^. Interpersonal distance has the potential to reveal our emotional and mental attitudes towards others. Apart from our own emotional states, the emotions that we perceive in others are also crucial in determining our approach and avoidance behaviors. Typically, mentally healthy individuals tend to favor positive self-referential processing^16,17^, a preference that may protect against internalizing disorders such as depression^18^.

At the neural level, several cortical areas have been described as the neural structures of self-other discrimination. Neuroimaging studies found the role of the right superior marginal gyrus (SMG), as a part of the right temporo-parietal junction (TPJ), in self-other distinction^19,20^. The posterior portion of the right TPJ involves in non-affective self-other distinction^21,22^. The potential rationale behind this function of the rTPJ could be elucidated by considering its fundamental roles, which encompass spatial perception^23^, mental rotation^24^, the representation of distinct self and other aspects^25^, and inhibitory processes that prioritize the consideration of others over the self^26^. Furthermore, the ventromedial prefrontal cortex (vmPFC) is involved in both self and other perception^27^. While the vmPFC is involved in both self and other perception and shows some overlap in the brain structure, the area responsible for self-perception tends to be located more ventrally, whereas the other-related area is typically more dorsal^27,28^. Sui and Humphreys^29^ in an fMRI study described the activation of the vmPFC in the processing of self-related stimuli, while the dlPFC activated in the processing of the stranger-related stimuli^30^. Humphreys and Sui propose a self-attention network in which the vmPFC is a self-representation core, which is controlled by the dlPFC through a top-down control^31^.

Beyond correlational neuroimaging studies, non-invasive brain stimulation (NIBS) studies found causal association between these cortical areas and self-other distinction. Transcranial magnetic stimulation (TMS) found the role of the right SMG^19,32^ and the vmPFC^33^ in self-other distinction. Transcranial direct current stimulation (tDCS) also described the role of the right TPJ and the vmPFC in self-other discrimination. For instance, anodal right TPJ stimulation improved self-other representations^26,34^, facilitates embodied mental rotation of the self into an alternate perspective^35^ and increased the effect of bodily position during perspective-taking^36^. Anodal vmPFC stimulation improves social framing^37^, self-referential processing^38^, and altruistic behaviors^39^. Several tDCS studies found that the vmPFC as a part of off-task/default mode network (DMN) work in concert with the dorsolateral prefrontal cortex (dlPFC) as the hub of on-task/central executive network (CEN) in several social/emotional processing, including emotional processing^40^, self-referential processing^38^, and emotion regulation^41^. However, a HD-tDCS study found a null effect of the dlPFC and the vmPFC in prioritization effects for self, friend, and stranger^42^.

tDCS represents a non-invasive technique for modulating the brain's excitability and activity patterns^43,44^. Employing tDCS involves the application of a gentle electrical current through paired anodal and cathodal electrodes, inducing upregulation and downregulation effects on the cortical regions beneath^44,45^. In the present study, we harnessed anodal tDCS to enhance the activity of the vmPFC, rTPJ, and dlPFC, aiming to assess its impact on interpersonal comfort distance and emotional approach/avoidance.

In summary, the vmPFC and rTPJ collaboratively serve as neural architects shaping social cognition by interweaving self-awareness and perspective-taking. This synergy aids our navigation of intricate social interactions^46^. Their coordinated functions underlie a spectrum of cognitive processes, spanning self-referential processing, theory of mind, and empathic connection^47,48^. This synthesis fosters a comprehensive grasp of self and others within our social milieu. Moreover, the dlPFC involvement in emotional processing and its regulatory influence on the vmPFC establish it as a significant contributor to the social cognition^40,49–51^. Considering the established significance of the vmPFC, rTPJ, and dlPFC in self-other differentiation and their implications for processing emotional content, our study endeavors to unravel their intricate roles in perceiving relationships to the self (self, mother, president) and in shaping the calibration of one's comfortable interpersonal space. Furthermore, we seek to delve into the nuanced influence of these neural structures on approach and avoidance responses concerning emotional faces, delineating their modulation in accordance with the valence of the emotional states at play.

## Materials and methods

### Participants

The study recruited twenty healthy adults (11 females) aged between 27 and 45 years, with an average age of 37.30 ± 5.47 years. Utilizing G*power calculations^52^, and employing a power of 0.95, significance level of 0.05, and considering a medium effect size (f = 0.40) recommended for tDCS studies^53^, our study's design, coupled with a repeated measures ANOVA, indicated a requisite sample size of 16 participants. Additionally, we included 4 extra participants to account for unforeseen situations. All participants were right-handed and had normal or corrected-to-normal vision, with no history of head trauma, drug addiction, or psychiatric or neurological disorders. The research was conducted in compliance with the ethical standards outlined in the Helsinki Declaration of 1975, as revised in 2013, and was approved by the national ethical committee (Ethics Code: IR.SBU.REC.1401.106).

### Comfortable interpersonal distance task (CIDT)

This task was used to measure the preferred interpersonal distance^13^. The test has been found to be valid and reliable in various cultures and correlates with optimal interpersonal distances in real-life interactions^9^. This task measures the optimal physical distance between oneself and others, and involves participants imagining themselves in a room with a circle drawn on paper.

**Figure 1:**
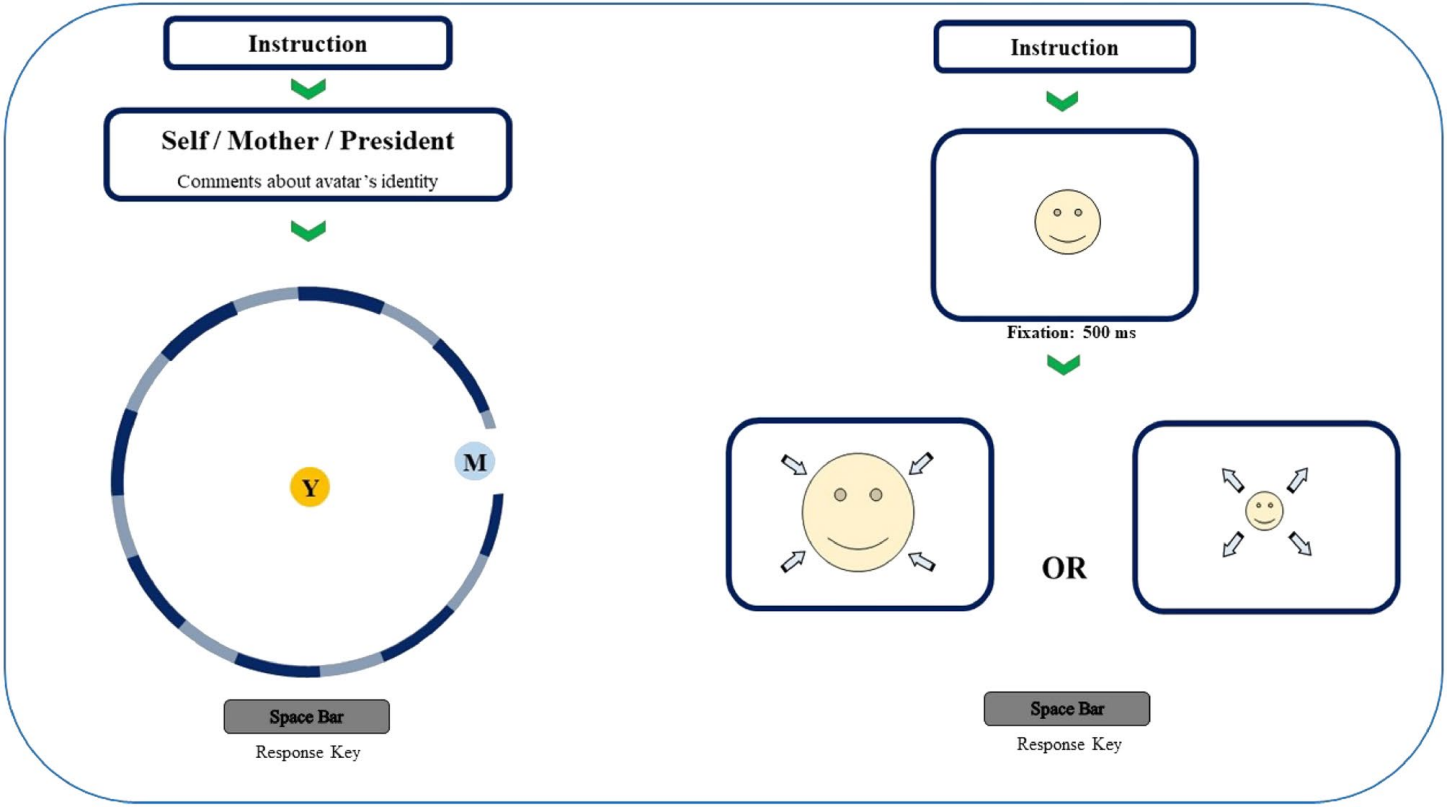
A diagrammatic representation of comfortable interpersonal distance task (CIDT, on the left) and the approach avoidance task (AAT, on the right).

In this study, a computerized iteration of the CIDT was deployed, integrating a 15 cm diameter circle displayed on the screen to simulate a room featuring eight doors. Within this virtual scenario, participants were guided to envision themselves stationed at the room's center. Across each trial, a dynamic avatar symbolizing distinct entities—namely, the self, the mother, or the president—would ingress in a random order through one of the doors and move toward the center of the room. Each participant was expressly informed with a written instruction and comment about the identity of the avatar before and during each trial. Across ten iterations for each avatar category, participants were tasked with gauging their comfort threshold and subsequently prompted to press the space key, effectively arresting the avatar's motion when they believed the interpersonal space had attained an optimal comfort level. This iterative process was executed for all three avatar identities, generating a dataset consisting of the distance in pixels from the center, a quantitative metric encapsulating the preferred interpersonal boundary. This distance metric ranged from 0 to 1000 pixels and encapsulated the outcomes of each trial, thereby serving as a pivotal measure of interpersonal comfort for the respective avatar categories, Fig. 1. The task took around 5 min to perform.

### Approach avoidance task (AAT)

This task was used to discover the proper distance with emotional material^54^. This task involves presenting an emotional stimulus on a screen that moves towards or away from the viewer, allowing the viewer to control its distance by pressing the space bar to stop its movement at a comfortable distance. During each trial, an image is displayed at the center of the screen, occupying half its size. After a 500 ms, the image progressively enlarges or diminishes, symbolizing its approach or withdrawal from the viewer until it covers the entire screen (100%) or vanishes (0%). By pressing the space bar, participants halt the image's movement, signifying approach or avoidance. The image size, represented as a percentage, quantifies the degree of approach or avoidance, Fig. 1. In this study, we used 100 emotional faces, happy, sad, angry, disgust and neutral faces, equally. The faces were selected from the NimStim set of facial expressions^55^. Stimuli of all tasks were presented on a laptop with a 15.2″ screen at a viewing distance of approximately 50 cm. The task took around 5 min to perform.

### tDCS protocol

**Figure 2:**
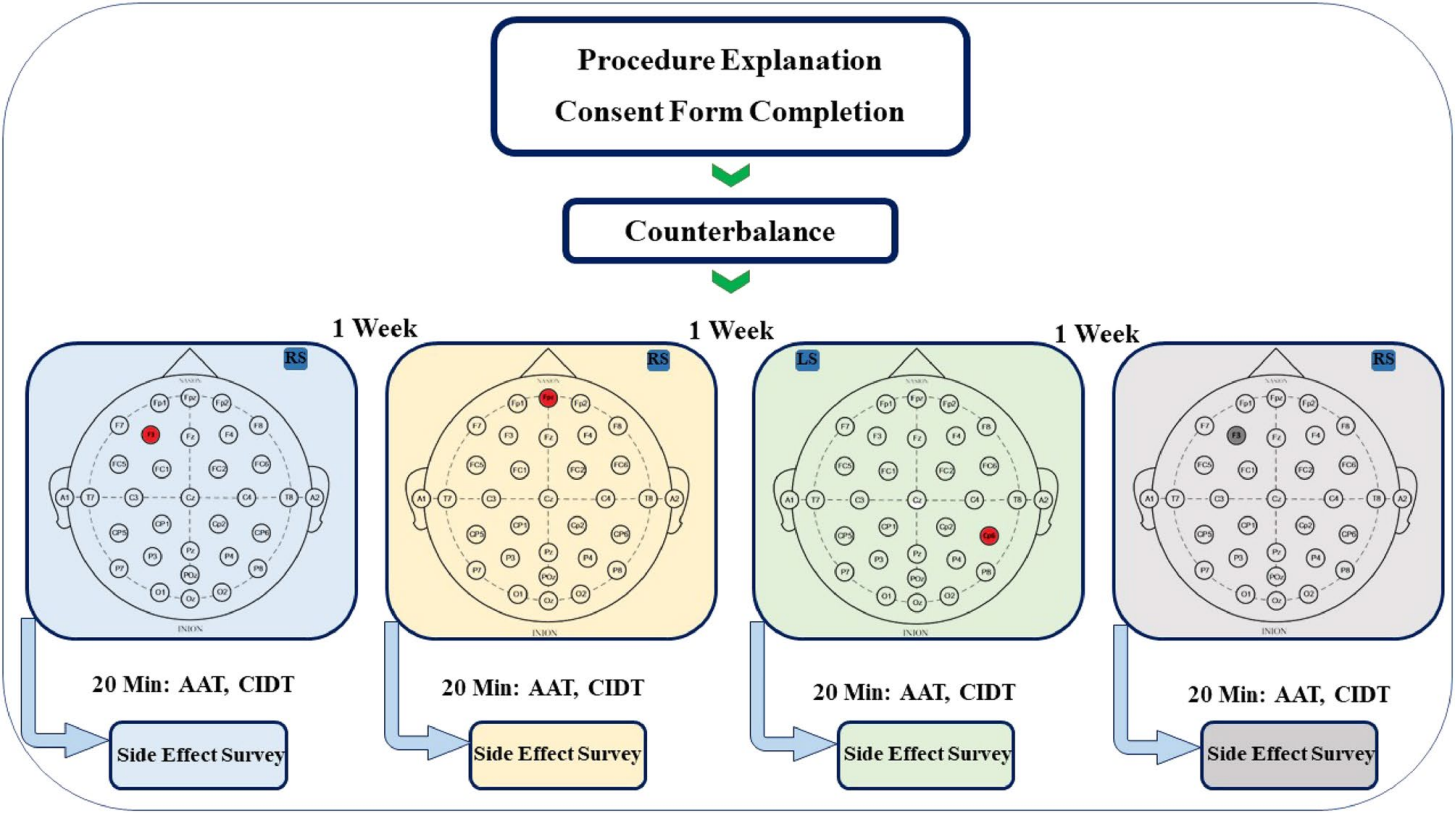
Experimental procedures. Participants received one of the tDCS protocols in randomized order. The AAT and CIDT were performed in a random order in each session 5 min after stimulation. Finally, the participants completed the side effect questionnaire. *CIDT* comfortable interpersonal distance task, *AAT* approach avoidance task.

**Figure 3:**
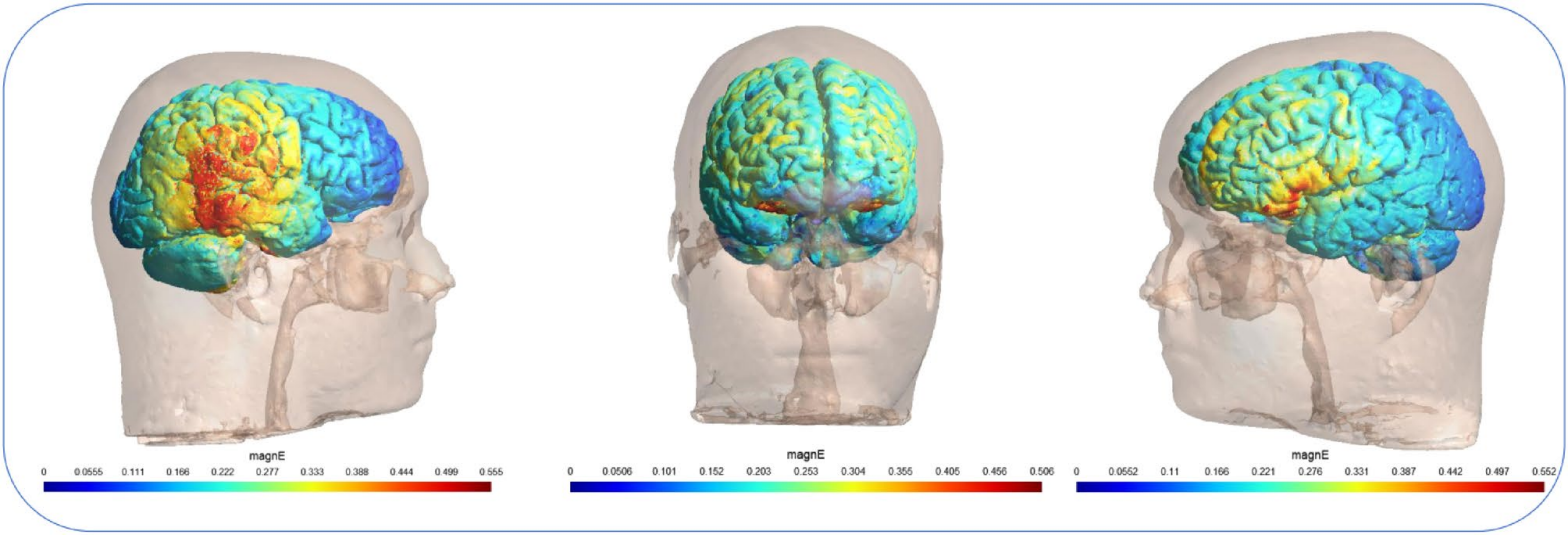
Distribution of electrical field calculated using SimNIBS. Two 5 × 5 cm electrodes were positioned over CP6 (left), FPZ (middle) and F3 (right) and the current intensity was set to 2 mA.

ActivaDose stimulator (ActivaTek Inc., USA) was used to deliver the stimulation. The stimulator produced an electrical direct current of 2 mA, which was applied for 20 min through a pair of saline-soaked sponge electrodes measuring 25 cm^2^ (5 × 5). The current was ramped up over a period of 30 s and down over a period of 30 s. The tDCS protocol involved four sessions, spaced one week apart, with the electrodes positioned according to the 10–20 EEG international system. The four conditions were: (1) anodal dlPFC (F3), (2) anodal vmPFC (FPZ), (3) anodal rTPJ (TP6), and (4) sham stimulation, Fig. 2. For the sham condition, the placement of the electrodes was randomly selected from one of the real conditions. The return electrode was placed on the contralateral shoulder in all conditions. Modeling the flow of electrical current to examine the electric field distribution using SimNIBS is illustrated in Fig. 3.

### Procedure

**Figure 4:**
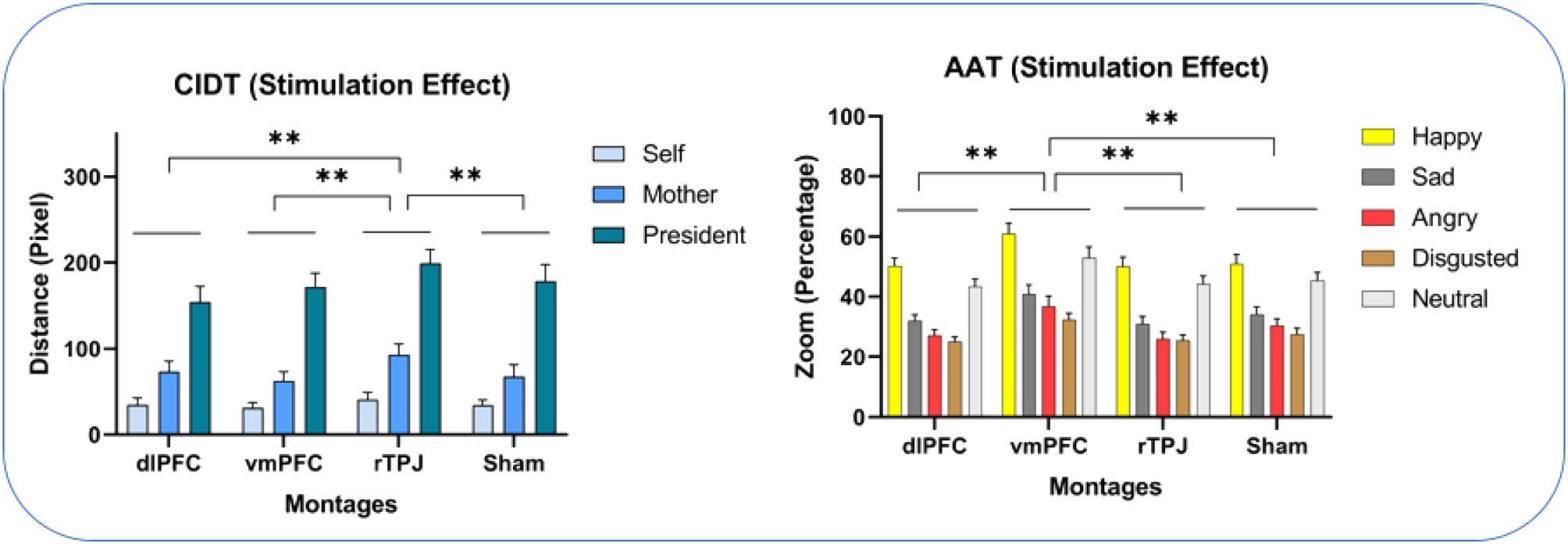
Shown are the effects of tDCS on the outcome measures. The vertical axis indicates the respective outcome measures of the tasks. The bars are showing the means, error bars represent standard error of means. The horizontal axes are showing stimulation conditions. *CIDT* comfortable interpersonal distance task, *AAT* approach avoidance task, *dlPFC* dorsolateral prefrontal cortex, *vmPFC* ventromedial prefrontal cortex, *rTPJ* right tempro-parietal junction.

Once the inclusion criteria were verified, and informed consent was obtained, the tasks and procedures were explained to the participants by AM. Then, in a single-blinded design, the stimulation was applied randomly and in a counterbalanced order. After five minutes of stimulation, the participants performed the AAT and ICD tasks in a random order, which took approximately 15 min, Fig. 4. Following each session, the participants completed a side effect checklist^56^ and were asked to guess whether they received real or sham stimulation.

### Data analysis

Data analysis was carried out using the statistical package SPSS for Windows. The assumptions of normality and sphericity were confirmed based on the outcomes of the Shapiro–Wilk and Mauchly's tests. Two repeated measures analysis of variance (ANOVAs) were conducted, with emotional state in AAT (happy, neutral, sad, angry, and disgusted) or avatar (self, mother, and president) and tDCS conditions (four conditions) serving as the within-subject factors, and the pixel of comfortable distance being adopted as the dependent variable. Mauchly's test was employed to assess data sphericity, and corrections to the degrees of freedom were made using the Greenhouse–Geisser method when necessary. Post hoc analyses utilized Fisher's Least Significant Difference (LSD) test. In addition, the incorporation of session order and task order as covariates within an ANCOVA was implemented. A significance level of p < 0.05 was applied to all statistical comparisons.

## Results

The participants tolerate the stimulation with minimal side effects. Table 2 displays the means and standard deviations of the different side effects across the stimulation conditions. The results of one-way ANOVAs indicated that there were no significant differences between the conditions for most of the side effects, except for burning (F_1.93_ = 6.15, p = 0.05, ηp^2^ = 0.24) and tingling (F_2.11_ = 3.65, p = 0.03, ηp^2^ = 0.16), Table 1. Post hoc LSD analysis revealed that during tDCS over dlPFC, vmPFC, and rTPJ, participants experienced higher levels of burning compared to sham stimulation (MD = 0.75, p < 0.001; MD = 0.55, p = 0.02; MD = 0.90, p < 0.001 respectively). Furthermore, the participants reported higher levels of tingling during stimulation of dlPFC (MD = 0.70, p = 0.03) and rTPJ (MD = 0.80, p < 0.001) relative to the sham condition. Guesses of real stimulation for vmPFC, dlPFC, rTPJ, and sham stimulation were 75%, 60%, 50%, and 40%, respectively (χ^2^(1) = 2.86, p = 0.09).

**Table 1:** Side effects of tDCS (means and sd) in the different stimulation conditions and the results of the respective ANOVAs.

Measures	Conditions, M(SD)				Statistics			
	dlPFC	vmPFC	rTPJ	Sham	df	F	P	ηp^2^
Pain	0.05 (0.22)	0.05 (0.22)	0.00 (0.00)	0.25 (0.44)	1.74	3.12	0.06	0.14
Vertigo	0.00 (0.00)	0.05 (0.22)	0.00 (0.00)	0.00 (0.00)	3	1.00	0.40	0.05
Burning	0.75 (0.71)	0.55 (0.99)	0.90 (0.85)	0.00 (0.00)	1.93	6.15	0.05	0.24
Tingling	0.80 (1.32)	0.45 (0.75)	0.90 (0.78)	0.10 (0.30)	2.11	3.65	0.03	0.16
Confusion	0.05 (0.22)	0.20 (0.52)	0.10 (0.30)	0.15 (0.36)	3	0.54	0.65	0.02
Drowsiness	0.25 (0.71)	0.55 (0.82)	0.80 (1.43)	0.70 (1.26)	3	1.01	0.39	0.05

*M* mean, *SD* standard deviation, *dlPFC* dorsolateral prefrontal cortex, *vmPFC* ventromedial prefrontal cortex, *rTPJ* right tempro-parietal junction. *df* degrees of freedom, *F* F-value, *P* P-value, *ηp*^*2*^ partial eta squared.

For the CID task, the ANOVA revealed a significant main effect of tDCS conditions (F_3_ = 6.94, p < 0.001, ηp^2^ = 0.26). Additionally, significant main effects were found for avatar identity (F_2_ = 48.45, p < 0.001, ηp^2^ = 0.71) and the interaction between stimulation and avatar (F_3.26_ = 2.71, p = 0.04, ηp^2^ = 0.12). The LSD post hoc analysis showed that during rTPJ stimulation, participants kept avatar images at a greater distance compared to dlPFC (MD (mean difference) = 23.53, p = 0.001), vmPFC (MD = 22.47, p = 0.001), and sham (MD = 17.49, p = 0.004) stimulation. For avatar identity, participants tended to keep the "president" stimuli at a further distance than the "self" (MD = 14.57, p < 0.001) and "mother" (MD = 101.89, p < 0.001) conditions. Furthermore, the "mother" stimuli were kept at a longer distance than the "self" stimuli (MD = 38.68, p = 0.002). The post hoc analysis of the interaction showed that during "mother" trials, distance scores were higher during rTPJ stimulation compared to dlPFC (MD = 19.51, p = 0.01), vmPFC (MD = 3.25, p = 0.001), and sham (MD = 25.37, p = 0.003) conditions. For "president" stimuli, larger distances were recorded under rTPJ stimulation compared to dlPFC (MD = 45.04, p = 0.004), vmPFC (MD = 27.41, p = 0.003), and sham (MD = 2.72, p = 0.01) stimulation, Tables 2 and 3.

**Table 2:** Mean and standard deviation of measurements in different stimulation conditions.

Measures	dlPFC	vmPFC	rTPJ	Sham
CIDT				
Self	34.69 (34.94)	30.99 (28.28)	40.74 (35.84)	34.34 (25.77)
Mother	73.14 (53.05)	62.40 (46.06)	92.66 (57.58)	67.28 (6.90)
President	154.02 (81.38)	171.65 (71.72)	199.06 (74.27)	178.34 (85.79)
Mean	87.28 (43.22)	88.35 (34.83)	110.82 (37.15)	93.32 (43.77)
AAT				
Happy	50.24 (11.28)	61.02 (15.89)	50.05 (13.89)	37.66 (8.98)
Neutral	43.35 (11.23)	52.89 (16.76)	44.19 (11.91)	45.34 (12.14)
Sad	32.04 (8.75)	40.96 (13.10)	31.02 (1.85)	50.85 (14.11)
Angry	27.22 (8.69)	36.82 (15.20)	26.06 (1.14)	30.45 (1.09)
Disgusted	25.15 (6.93)	32.36 (9.68)	25.53 (8.09)	27.53 (8.89)
Mean	35.60 (6.32)	44.81 (12.15)	35.37 (9.08)	93.32 (43.77)

*CIDT* comfortable interpersonal distance task, *AAT* approach avoidance task, *dlPFC* dorsolateral prefrontal cortex, *vmPFC* ventromedial prefrontal cortex, *rTPJ* right tempro-parietal junction.

**Table 3:** The result of two factorial ANOVA on the study measures.

	Stimulation effect				Avatar/emotion effect				Stimulation × avatar/emotion			
	df	F	P	ηp^2^	df	F	P	ηp^2^	df	F	P	ηp^2^
CIDT	3	6.94	< 0.001	0.26	2	48.45	< 0.001	0.71	3.26	2.71	0.04	0.12
AAT	3	7.21	< 0.001	0.27	1.94	5.92	< 0.001	0.72	5.19	0.99	0.42	0.05

*CIDT* comfortable interpersonal distance task, *AAT* approach avoidance task, *df* degrees of freedom, *F* F-value, *P* P-value, *ηp*^*2*^ partial eta squared.

For the AAT, the ANOVA results revealed significant main effects of stimulation conditions (F_3_ = 7.21, p < 0.001, ηp^2^ = 0.27) and emotion (F_1.94_ = 5.92, p < 0.001, ηp^2^ = 0.72). However, there was a non-significant interaction of stimulation and emotion (F5.19 = 0.99, p = 0.42, ηp^2^ = 0.50). Post-hoc analyses were performed using LSD to examine pairwise comparisons between tDCS conditions. The results showed that anodal vmPFC led participants to magnify emotional images more and keep them in a smaller distance than dlPFC (MD = 9.21, p = 0.003), rTPJ (MD = 9.44, p = 0.004), and sham (MD = 7.15, p = 0.005) stimulation. Furthermore, the LSD results indicated that participants tended to make emotional faces bigger in a descending order during happy, neutral, sad, angry, and disgusted trials. Specifically, there were significant differences between happy and sad (MD = 18.49, p < 0.001), happy and angry (MD = 22.90, p < 0.001), happy and disgusted (MD = 25.39, p < 0.001), happy and neutral (MD = 6.59, p = 0.01), neutral and sad (MD = 11.90, p < 0.001), neutral and angry (MD = 16.31, p < 0.001), neutral and disgusted (MD = 18.80, p < 0.001), sad and angry (MD = 4.40, p < 0.001), sad and disgusted (MD = 6.89, p < 0.001), and angry and disgusted (MD = 2.49, p = 0.01), Tables 2 and 3. ANCOVA indicated that there was no statistically significant effect attributable to task and session order.

In sum, the CID task indicated that anodal rTPJ stimulation led participants to maintain greater distance compared to other stimulation conditions. Notably, the "president" avatar was kept at a further distance than both the "self" and "mother" avatars. Moreover, during "mother" trials, distances were greater under rTPJ stimulation compared to "self" trials. In the AAT, anodal vmPFC stimulation resulted in participants magnifying emotional faces, independent of emotional states, more and keeping them at a smaller distance compared to other stimulations.

## Discussion

In this study, our objective was to evaluate the effects of stimulating three brain regions—the dlPFC, the vmPFC, and the rTPJ—on self-other discrimination and approach/avoidance behavior concerning distinct identities of others and varied emotional states. Independent of others identity and emotional states, anodal stimulation of the rTPJ has a significant impact on self-other distance, while anodal stimulation of the vmPFC increases approach behavior. Additionally, the identity of avatar has a significant impact on the effectiveness of anodal rTPJ stimulation, the comfortable distance was larger for president and mother. However, the vmPFC plays a valence-insensitive role in emotional processing. In the following section, we will discuss the results in the context of previous brain stimulation studies.

### Increased interpersonal comfortable distance

The results suggest a longer interpersonal comfortable distance to mother and president, and not the self, during anodal rTPJ stimulation compared to all other conditions. Earlier tDCS studies found improved self-other discrimination during anodal right rTPJ stimulation using video-morphing task^26^, perspective taking task^34^. Uddin et al. observed that applying rTMS to the right inferior parietal lobule (IPL), which is part of the temporoparietal area, resulted in a disturbance in the capacity to differentiate one's own face from that of another^20^. Neuroimaging studies have revealed that the rTPJ plays a key role in various complex social cognitive functions such as theory of mind^57^, empathy^58^, and perspective taking^59,60^. Interestingly, these complex social-cognitive functions also involve basic self-other processing. The areas of the brain responsible for self-other processing overlap with those associated with complex social-cognitive functions and include the rTPJ^4^.

In the present study, the distance of self and other was greater during anodal rTPJ, indicating an egocentric role of the rTPJ. A tDCS study found anodal rTPJ increase self-effacing attributions, refers to downplay the self-role in positive outcomes instead of external factors^61^.

Similarly, several tDCS studies found anodal rTPJ stimulation did not improve ToM functioning in healthy adults^62,63^ However, one study did observe a decrease in ToM accuracy following cathodal stimulation of the rTPJ^64^. It seems the rTPJ plays a perceptual/physical role in social cognition, including theory of mind, refers to the ability to mentally rotate into an allocentric viewpoint, known as perspective taking, is considered a prerequisite for ToM^65^. This role of the rTPJ in lower-order, but not higher order, social processing has been described earlier^35^.

The interaction between stimulation and avatar identity revealed the role of the vmPFC for increase the interpersonal comfortable distance for the president and mother avatars, but not the self-avatar.

Notably, the notion of self-other distinction, emblematic of the capability to delineate self from other, underscores that moving the self closer to itself does not represent self-other distinction; rather, it can be perceived as a control condition.

### Increased approach to emotional stimuli

Independent of the emotional expressions, anodal vmPFC increased approach to emotional stimuli. Earlier tDCS studies found application of anodal tDCS over the vmPFC with an extracranial reference electrode increased attention bias to happy faces or scenes, while cathodal tDCS decreased this bias^66^. Another tDCS study described an attenuation of anger and aggression during anodal vmPFC stimulation using anger-infused ultimatum game in healthy participants^67^. Moreover, anodal vmPFC stimulation with extracranial reference electrode enhanced pleasant scene processing in healthy participants^68^. These results are in further accordance with evidence from neuroimaging studies showing that the vmPFC downregulates emotionally negative responses of the amygdala^69,70^. Regardless of the montage used, individuals tend to respond more to positive facial expressions than negative ones. In the present study, we did not observe any significant interaction between expressions and stimulation. This result can be explained by the role of the ventromedial prefrontal cortex (vmPFC) in emotional processing. An earlier tDCS study in healthy participants showed that the vmPFC plays a role in arousal, but not valence, of emotional stimuli^40^. However, Iarrobino et al. conducted a crossover study using anodal stimulation on the right and left vmPFC, with an extracranial return electrode, and found that discrimination of angry faces increased with left-sided stimulation, while discrimination of sad faces decreased with right-sided stimulation in healthy individuals^71^. This study used an explicit emotion recognition task, which is relatively different from the approach-avoidance test used as an implicit task in the current study.

### Limitation and future direction

There are certain limitations to this study that need to be considered. Firstly, the study had a single-session design to explore the role stimulation of some target areas in self-other discrimination and approach, which may limit the ability to draw a conclusion about the clinical relevance of the intervention. Furthermore, we target the dlPFC, vmPFC and rTPJ as involved area in self-other discrimination. However, the involves brain areas in self-other perception and discrimination are not limited to these areas. Secondly, the sample size of this pilot study was moderate and the design was single-blind. For future studies, a larger sample size and a double-blind design should be implemented to improve the rigor of the design. Furthermore, future studies should include physiological markers to understand the underlying mechanisms of the observed cognitive effects and titrate the intervention dosage according to intensity and duration for personalized intervention approaches.

## Conclusion

This study revealed that the rTPJ plays a critical role in self-other discrimination, particularly in situations where the moving avatar represented mother or president, and not for the hypothetical self. These results suggest that the rTPJ is involved in self-other discrimination. On the other hand, the study revealed that anodal stimulation of the vmPFC increases approach behavior towards positive emotional stimuli, indicating its involvement in emotional component of approaching. In summary, the vmPFC can be considered the emotional component of self-referential processing, while the rTPJ plays a perceptual role in self-other distinction.

## Data Availability

The datasets generated during the current study are available from the corresponding author on reasonable request.
